# Enhancing *De Novo* Transcriptome Assembly by Incorporating Multiple Overlap Sizes

**DOI:** 10.5402/2012/816402

**Published:** 2012-04-23

**Authors:** Chien-Chih Chen, Wen-Dar Lin, Yu-Jung Chang, Chuen-Liang Chen, Jan-Ming Ho

**Affiliations:** ^1^Department of Computer Science and Information Engineering, National Taiwan University, No. 1, Sec. 4, Roosevelt Rd., Taipei 10617, Taiwan; ^2^Institute of Plant and Microbial Biology, Academia Sinica, 128 Academia Road, Section 2, Nankang, Taipei 115, Taiwan; ^3^Institute of Information Science, Academia Sinica, 128 Academia Road, Section 2, Nankang, Taipei 115, Taiwan

## Abstract

*Background*. The emergence of next-generation sequencing platform gives rise to a new generation of assembly algorithms. Compared with the Sanger sequencing data, the next-generation sequence data present shorter reads, higher coverage depth, and different error profiles. These features bring new challenging issues for *de novo* transcriptome assembly. *Methodology*. To explore the influence of these features on assembly algorithms, we studied the relationship between read overlap size, coverage depth, and error rate using simulated data. According to the relationship, we propose a *de novo* transcriptome assembly procedure, called Euler-mix, and demonstrate its performance on a real transcriptome dataset of mice. The simulation tool and evaluation tool are freely available as open source. *Significance*. Euler-mix is a straightforward pipeline; it focuses on dealing with the variation of coverage depth of short reads dataset. The experiment result showed that Euler-mix improves the performance of *de novo* transcriptome assembly.

## 1. Introduction

With the rapid development of next-generation sequencing technologies, studies on genomics and transcriptomics are moving into a new era. However, while these new technologies produce a great quantity of highly accurate sequences, they also have a major drawback, in that most of these efficient technologies produce shorter read lengths. For instance, technologies based on cyclic reversible termination [1] and ligation-based sequencing [2] produce read lengths ranging from 15 bps to 125 bps. These lengths are sufficient for resequencing, but challenging for *de novo* assembly. In response to this problem, several new assemblers that are designed for short reads have recently been introduced. They can be divided into three categories: (1) greedy extension approaches, such as SSAKE [3], VCAKE [4], and SHARCGS [5]; (2) overlap-layout-consensus approaches, such as Edena [6]; (3) Euler-path approaches, such as Velvet [7], EULER-SR [8], AllPATHS [9], and ABySS [10]. Among them, Euler-path approaches seem more appropriate for processing large amount of short reads [10], because they use *k-*mer hashing to detect overlaps at less computational costs compared to traditional overlap-layout-consensus approaches. Recently, research works on Euler-path approaches have focused on both error removal and repeat resolution for genomic sequences, whereas only a few works shed light on *de novo* transcriptome assembly [11, 12]. However, *de novo* transcriptome assembly offers a unique opportunity to study the metabolic states of organisms [12] and provides an alternative path to study nonmodel organisms [13] and thus is a desirable and challenging approach. The main difference between genome assembly and transcriptome assembly is the variation of coverage depth. For example, in a genome assembly project, short reads are randomly sampled from a genome, and thus the coverage depth is anticipated to be uniformly distributed on the genome. On the other hand, the distribution of short reads in a transcriptome analysis project is highly dependent on gene expression levels, and the abundance of expressed genes exhibits a power-law distribution [14]. While the coverage depth is related to the key parameter *k* (or *k*-mer size) for Euler-path approaches [7, 15], it seems that a single run of an Euler-path approach program would not be sufficient for a *de novo* transcriptome assembly project. In this paper, we study the relationships between sequencing error rate, coverage depth, and parameter *k* using simulated data. Accordingly, we propose a transcriptome assembly procedure, called Euler-mix, for *de novo* assembly of whole transcriptome shotgun sequencing data. The primary innovation of Euler-mix is to utilize the relationship between parameter  *k* in Euler-path approaches and the coverage depth of sequence data. Finally, we demonstrate the performance and practicability of the proposed procedure, using a real transcriptome dataset of mice.

## 2. Results

### 2.1. On the Relationship between Coverage Depth and Optimum k

**Figure 1:**
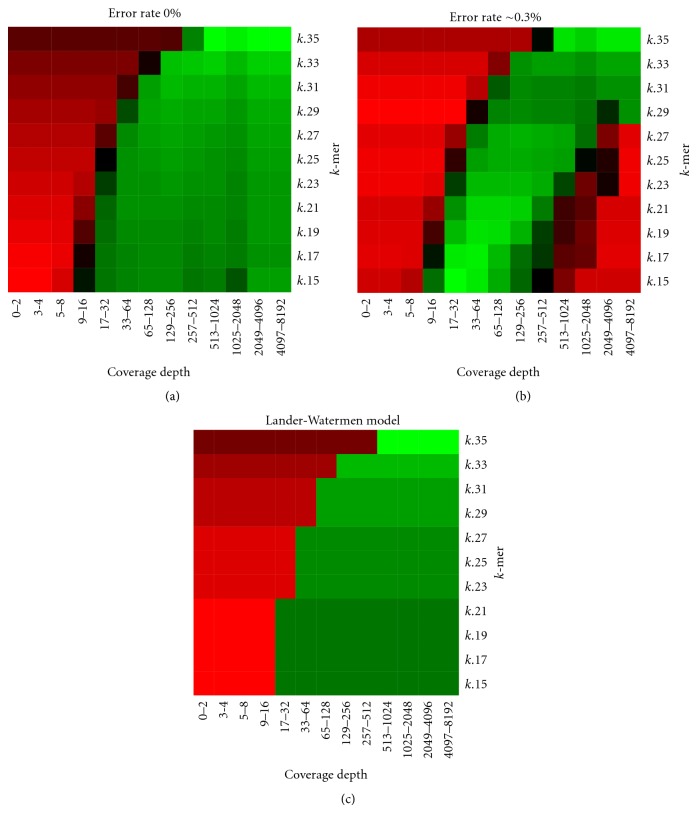
The relationship between optimum *k* and coverage depth.

For genome assembly projects, it has been shown that the parameter *k* of Euler-path approaches affects assembly results and is related with coverage depth [7]. Because the coverage depths of transcripts are correlated with expression levels and are thus varied, it is necessary to study the relationship between coverage depth and *k*'s that optimize assembly. To do this, we conducted an experiment on two simulated transcriptome datasets of mice, one is error-free and the other is with a sequencing error rate ~0.3%. Each dataset is composed of 80 million pair-ended 36 bp reads, and the range of transcript coverage depths extends from 1 to 4266, where coverage depths are proportional to corresponding expression levels that were computed from all mouse libraries in the NCBI dbEST database. We separately assembled each transcript by Velvet with different parameter *k*'s, and a *k* value was classified as optimum for a transcript if the consistent recall rate (Section 4) of the transcript was above 95%. Figures 1(a) and 1(b) show the relationship between optimum *k* and coverage depth for the error-free dataset and the error-rate-0.3% dataset, respectively. Here, a red-green heat map was used to indicate the degree of optimization: a green cell represents a higher ratio of transcripts that achieve 95% consistent recall rate and a red cell represents a lower ratio of these transcripts. From these figures, we observed two phenomena. First, upper left corners of Figures 1(a) and 1(b) are red, which means that optimum *k*'s of transcripts of lower coverage depths are distributed on smaller values. On the other hand, the lower right corner of Figure 1(b) is red, which implies that optimum *k*'s of transcripts of higher coverage depths are distributed on larger values when there is a sequencing error rate.

Since the meaning of *k* can be treated as the minimum length of overlap for two short reads to form a longer contig, the lower coverage depth implies less chance to have an overlap longer than or equal to *k*. This means shorter contigs and furthermore explains why a smaller *k* is more suitable for transcripts of lower coverage depths. Unsurprisingly, the well-known Lander-Waterman model [16] explains this first phenomenon. In their model, the expected number of contigs in a genome assembly project is (*c* ^*^ *G*/*L*)  *e* ^−(1−(*k*/*L*))*c*^, where (1) *G*  = genome length; (2) *L* = read length; (3) *c* = coverage depth; and (4) *k* = minimum length required for the detection of an overlap. Let every transcript be the genome in the Lander-Waterman model. We used this formula to estimate the relationship between optimum *k*'s and coverage depth, where a *k* value was classified as optimum for a transcript if the expected number of contigs is less than or equal to 1. In Figure 3(c), we summarized the relationship between optimum *k*'s and coverage depth as what we did in Figures 3(a) and 3(b), and it showed high similarity between Figures 3(a) and 3(c) hence described the first phenomenon.

For the second phenomenon, it should have been due to the fact that sequencing errors would result in “tips” and “detours” in underlying de Bruijn graphs [7]. Although current Euler-path approaches have been designed to handle most of such undesirable cases, the sequencing error rate times the higher coverage depths means more erroneously called bases, which means more chances to produce longer tips and detours that would not be resolved. Additionally, a smaller *k*, compared to a larger *k*, would give more chances to produce tips and detours. Thus, using a larger *k* to assemble very high coverage depth data is a practical approach when there is a sequencing error rate.

### 2.2. The Effect of Sequencing Error Rate

**Figure 2:**
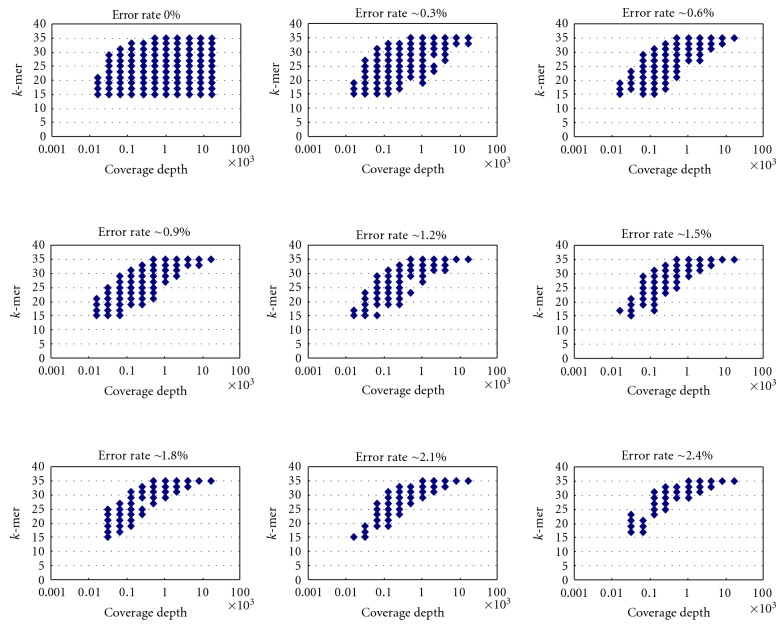
The relationship between optimum *k*'s and coverage depth for one transcriptome sequence in different error rate.

Because a sequencing error rate of 0.3% is commonly seen in the control lane of the Illumina Solexa sequencer [17], it is possible that the sequencing error rate might increase for noncontrol lanes. To see the crosstalks among coverage depth, sequencing error rate, and optimum *k*, we arbitrarily picked five mouse transcripts and generated simulated datasets with coverage depths 2x, 4x, 8x, 16x,…, and 16384x, respectively. Additionally, errors were simulated with average rates of 0%, 0.3%, 0.6%, 0.9%,…, and 2.4% for every coverage depth (Section 4).  Figure 2 shows results of one simulated transcript (see Figures S1–S4 in Supplementary Material available on line at doi:10.5402/2012/816402) for results of other four transcripts), which demonstrate a consistent trend with Figure 1(b). With the increased error rate, the range of optimum *k*'s of each coverage depth narrows and a positive correlation between coverage depth and optimum *k*'s becomes noticeable. It should be noticed that, for all datasets with sequencing errors, no *k* remains optimum for most tested coverage depths.

### 2.3. The Euler-Mix Assembly Procedure

Choosing an appropriate parameter *k* for Euler-path approaches is a practical issue for short read sequence data. From the above experiments, we see that coverage depth affects the distribution of optimum *k*'s and that no *k* is optimum for all coverage depths if there are sequencing errors. Thus, the issue of choosing *k* becomes tricky, especially for *de novo* transcriptome assembly, where data of different coverage depths are mixed in one sample. However, utilizing the correlation between coverage depth and optimum *k*'s, we can merge the results of different parameter *k*'s together and produce a more accurate assembly of transcriptome sequencing data.

**Figure 3:**
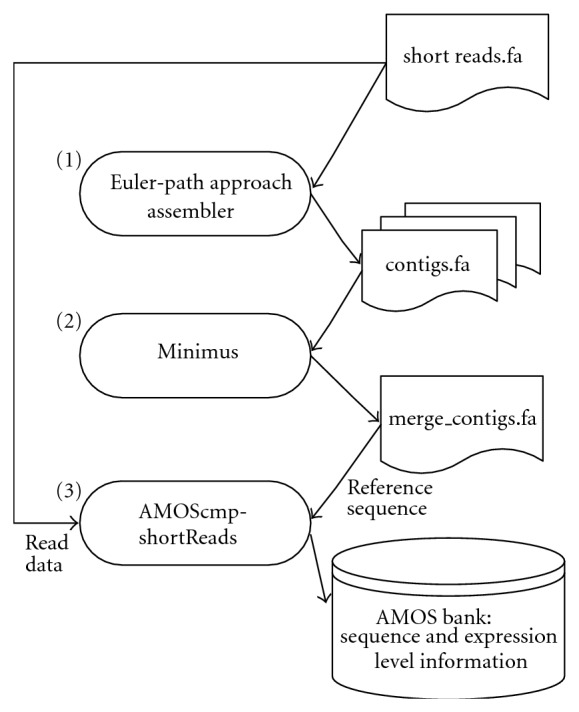
Overview of Euler-mix procedure.

To this end, we propose the Euler-mix procedure, which integrates existing assembly programs to deal with the varying coverage depth of transcriptome shotgun sequencing data. The Euler-mix procedure is based on two observations, (1) a larger *k* is suitable for data of higher coverage depth; while a smaller *k* is suitable for data of lower coverage depth and (2) the assembly result of an optimum *k* is similar with that of an adjacent optimum *k* in a range of coverage depth. Note that the second observation was because of the 95% consistent recall rate ensured by an optimum *k*. Figure 3 shows the overview of Euler-mix procedure.

The Euler-mix procedure contains three stages. For the initial stage, we assembled reads using an Euler-path approach. Since the coverage depth affects the selection of the parameter *k*, we applied all applicable *k*'s to assemble reads. We selected 19 as the smallest *k* and the length of reads as the upper bound of *k*. Then, we obtained different assembled results for the same input dataset. Because of the variation of coverage depths and applying multiple *k*'s, some results have better performance for transcripts of higher coverage depths, while others for transcripts of lower coverage depths. For the second stage, we use sequence assembly tools to assemble those results again using larger overlap size. This was done in order to join them together, because there could be duplications and overlaps between results by different *k*'s. In our experiment, we use Minimus [18] as the second stage's assembler with its default overlap size (*k* = 40). Minimus is a lightweight assembler, which is designed as a component of a larger assembly pipeline. It provides a systematic way to compute overlaps, identify uniquely assembled contigs, and use multiple sequence alignment to generate consensus sequences. These steps efficiently merge multiresults of an Euler assembler with different *k*'s into a more accurate file of contigs. For the third stage, we remap reads to the contig file using resequence tools, such as AMOScmp-shortReads [19]. After this third stage, we acquired a final assembly result with expression level information.

### 2.4. Comparing Euler-Mix with Existing Tools

To test the performance of Euler-mix and compare it with existing tools, we used the simulated dataset of the entire mouse transcriptome with a sequencing error rate of 0.3% (Section 4) as a benchmark. In the experiment, we used all *k*'s in three Euler-path approaches, including Velvet, EULER-SR, and ABySS, to compare with Euler-mix. For Euler-mix, we separately adopted aforementioned algorithms as the underlying assembly algorithm. Note that we processed the entire dataset at the same time, instead of processing each transcript one by one, because we were trying to mimic the actual application of the transcriptome assembly.  Table 1 shows the results on the simulated data executed by Velvet with *k* from 17 to 35, and by Euler-mix using Velvet as the underlying algorithm (see Supplementary Tables S1 and 2 for other algorithms). For overlap measures (Section 4), it shows that Euler-mix achieved the best precision, recall, and F-measure, where the recall rate was improved by about 5%. For the consistent measures (see materials and methods), it shows that Euler-mix achieved the second best precision (96.32%), whereas the best precision (96.59%) was achieved by Velvet with *k* = 17, whose recall was just 8.13%. With the best consistent recall, Euler-mix also achieved the best consistent F-measure. Compared with Velvet with best consistent F-measures (*k* = 21 and *k* = 23), Euler-mix improved consistent recall by more than 4%, reduced the number of contigs longer than or equal to 100 bps from 80,166 and 65,348 to 48,183 and extended the average size of contigs from 582 and 701 to 1,001. It shows that Euler-mix filled many of the gaps between highly fragmented contigs. Furthermore, the precision rate was higher than 90% no matter which underlying algorithm was applied, which implies that the quality of resulting contigs is reliable. Such enhancement also appeared when comparing Euler-mix with EULER-SR and ABySS.

### 2.5. A Transcriptome Assembly Application

To evaluate Euler-mix in real condition, we use a real data recently published by Trapnell et al. [20]. The data includes 430 million pair-ended 75-bp RNA-Seq reads from a mouse myoblast cell line over a differentiation time series. We selected the dataset in one time point (NCBI Short Read Archive, accession no. SRX017794, run SRR037945) which contains 44.37 million pair-ended 75 bp reads, to evaluate Euler-mix and Velvet with different *k* parameter.

In Euler-mix procedure, we performed Velvet with *k* from 21 to 75 on the dataset firstly. Considering the quantity of the results, we used Velvet in single-end mode with *k* = 39 as the second stage's assembler because Minimus is a light weight assembler which is not suitable for dataset too large. Note that the selection of *k* in the second stage's assembler is a trade-off between precision and recall (specificity and sensitivity).  Table 2 shows the experimental results of Euler-mix and Velvet with the best consistent F-measure (*k* = 33). Note that we used RNA sequences of M_musculus in NCBI RefSeq database as reference sequences for computation of all evaluation measures (see Materials and Methods). The number of transcripts detected defined here is the number of transcripts with overlap recall rate greater than or equal to 80%. Compared with Velvet with the best consistent F-measure, Euler-mix improved consistent F-measure by more than 8% and increased the number of transcripts detected from 2,544 to 10,646. This experiment result shows that Euler-mix has significant improvement in *de novo* transcriptome assembly in real case.

## 3. Discussion

In our experiments, we found that optimal parameter *k*'s of Euler-path assemblers are positively correlated with the coverage depth of the sequence data. This phenomenon is due to the fact that lengths of overlaps between the reads are highly dependent on the coverage depth and error rate. The lower the coverage depth, the lower the probability of having a longer overlap between reads. Thus, selecting a shorter minimum overlap as a criterion for assembling reads is more suitable for low coverage depth data. On the other hand, the higher coverage depth may amplify the occurrence of sequencing errors in reads. Therefore, selecting a longer minimum overlap as a criterion for assembling reads should filter out the noise for high coverage depth data.

As for transcriptome sequencing data, the coverage depths are associated with expression levels. Because the expression levels exhibit a power-law distribution, choosing an appropriate parameter *k* for Euler-path approaches becomes a problematic issue. However, these problems can be solved by taking all of the possible overlap sizes into account. Our experiments show that by merging the results of different *k*'s of Euler-path approaches, we shall obtain better performance for *de novo* transcriptome assembly.

Similarly, the same benefit also appears in the overlap-layout-consensus approach. We applied Edena [6] to assemble the simulated data with different overlap sizes, and compare them with the combined result merged by Minimus. It shows that the combined result reduces the number of contigs longer than or equal to 100 bps and extends average contig size, which suggests that combining results of different *k*'s improves the performance (see Supplementary Table S3).

In this paper, we also proposed consistent measures in addition to using only overlap measures (see materials and methods). One of the most important differences between these two kinds of measures is that consistent measures distinguish better assembly results from other assembly results. In most of our experiments, overlap measures gave precision higher than 99%, but consistent measures give precision values different from each other. In other words, consistent measures do provide a more accurate evaluation on the performance of assembly results.

Our next target is to investigate how to use mate-pair information properly in Euler-mix. Because most existing assemblers use mate-pair information that is aimed at repeat of genomic DNA and based on the assumption that short read data have uniform coverage depth, it is an interesting issue for transcriptome data to propagate the mate-pair information from first stage's assembler to the second stage's assembler correctly in Euler-mix and produce even longer and more accurate contigs.

## 4. Materials and Methods

### 4.1. Simulated Dataset

**Figure 4:**
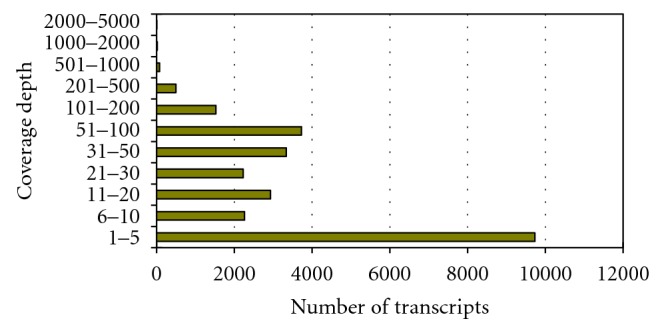
Histogram of the coverage depths (expression levels) of the 26,332 transcripts of mice.

We created a synthetic dataset that mimicked the experimental data of transcriptome shotgun sequencing. The synthetic dataset of 80 million pair-ended 36 bp reads was randomly sampled from 26,332 transcripts of mice, which were collected from the NCBI RefSeq database [21]. To mimic the varied coverage depth of transcriptome shotgun sequencing data, the number of reads of each transcript was proportional to the number of ESTs multiplied by the length of the transcript, where the EST numbers were computed according to the NCBI dbEST database. Most transcripts have low coverage depths, and the variation of coverage depth is large, extending from 1 to 4,266 (see Figure 4). Additionally, the distribution of the coverage depth is a power-law distribution and is similar to the experiment data of the whole transcriptome shotgun sequencing for HeLa [22]. To better fit in with real-world data, we applied error rates that were slightly increased from start to end in reads. For the average error rate of 0.3%, the error rate at first nucleotide is 0.2% and increased 0.005% for every next nucleotide. Similarly, for average error rates 0.6%, 0.9%,…, and 2.4%, the error rates start with 0.5%, 0.8%,…, and 2.3%, respectively. The sizes of inserts were uniformly distributed from 175 to 225.

### 4.2. Overlap Measures

To evaluate the performance of assembly results, we created overlap measures, which take the following steps. First, we used MegaBLAST [23] to align assembled contigs longer than or equal to 100 bps to reference sequences, and only alignments with at least 95% identity were taken into account. The union of all alignment areas in the reference was treated as true positives, and we computed the overlap recall rate using the following formula:

(1) $$\text{Recall}=\frac{\text{number}\;\;\text{of}\;\;\text{true}\;\;\text{positive}\;\;\text{bases}\;\;\text{in}\;\;\text{reference}}{\text{total}\;\;\text{length}\;\;\text{of}\;\;\text{reference}}.$$

Similarly, the union of all alignment areas on the contig side was treated as true positives in contigs, and the overlap precision rate was defined as

(2) $$\text{Precision}=\frac{\text{number}\;\;\text{of}\;\;\text{true}\;\;\text{positive}\;\;\text{bases}\;\;\text{in}\;\;\text{contigs}}{\text{total}\;\;\text{length}\;\;\text{of}\;\;\text{contigs}}.$$

By that, the weighted harmonic mean of precision and recall, F-measure, was defined as

(3) $$\text{F-measure}=\frac{2\times \text{precision}\times \text{recall}}{\text{precision}+\text{recall}}.$$

**Figure 5:**
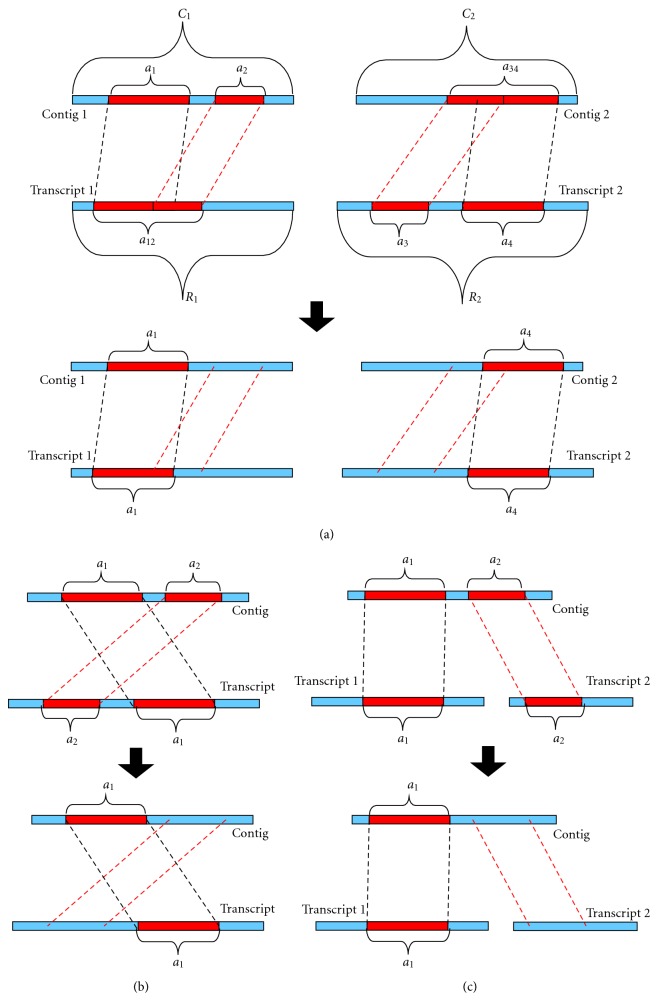
Illustration of overlap measures and consistent measures.

Figure 5(a) presents an example of how overlap precision and overlap recall were computed: the overlap precision rate is (*a*_1_ + *a*_2_ + *a*_34_)/(*C*_1_ + *C*_2_) and the overlap recall rate is (*a*_12_ + *a*_3_ + *a*_4_)/(*R*_1_ + *R*_2_). Note that the true positive area may include overlapping regions of alignments, so we named these measures overlap. Also note that these measures may overestimate the performance because of recounting the overlaps. However, most works use them as the benchmark, for example, the “sequence coverage” used in Velvet and the “genome coverage” used in ABySS. Accordingly, we take overlap measures as the upper bound of performance.

### 4.3. Consistent Measures

Theoretically, a perfect assembly result consists of exactly the same sequences as the reference sequences. Thus, in such a perfect result, every repetitive region is maintained and no alignment rearrangement is included. In order to distinguish better assembly results from those collapse repetitive regions or contain alignment rearrangements, we designed consistent measures, which exclude some alignments from counting the true positive area. For example, Figure 5(a) shows a case where alignments *a*_1_ and *a*_2_ overlap in the reference sequence; similarly, alignments *a*_3_ and *a*_4_ overlap in a contig. All these cases would amplify accuracy aversively if considered only under the overlap measures. To remedy this problem, we choose only one alignment from *a*_1_ and *a*_2_ as the true positive; in the same way, only one alignment was chosen from *a*_3_ and *a*_4_. As a result, the consistent precision rate is (*a*_1_ + *a*_4_)/(*C*_1_ + *C*_2_) and consistent recall rate is (*a*_1_ + *a*_4_)/(*R*_1_ + *R*_2_) in Figure 5(a).  Figure 5(b) shows a case where alignments *a*_1_ and *a*_2_ are rearranged. Since the assembly results with alignment rearrangements should be treated differently from those without alignment rearrangements, only one alignment is considered correct.  Figure 5(c) shows that one contig has two alignments from different transcripts. In the case of the transcriptome assembly, one contig should stand for only one transcript; thus only one alignment is chosen in Figure 5(c).

To follow all these aforementioned rules, the consistent measures were implemented according to the following steps.

**Step 1 .**

BLAST contigs against reference sequences; sort all alignments according to bit scores.

**Step 2 .**

For every alignment from highest bit score to lowest bit score, add an alignment into the list *Alignment Collection* (initially empty) if

2(a) it does not overlap with any alignment in *Alignment Collection* for more than 5% area in any side (the case of Figure 5(a)),
2(b) it is “consistent” with all alignments in *Alignment Collection* (the case of Figures 5(b) and 5(c)).

**Step 3 .**

Compute union area of all alignments in *Alignment Collection* for reference side, take this union area as true positive, and thus compute recall rate. Similarly, precision rate would be computed.

Note the true positive area in each contig is aligned with the same transcript, and thus the consistent precision rate represents the quality of an assembly result.

## Supplementary Material

Figures S1~S4 show the relationship between optimum *k*'s and coverage depth for four transcriptome sequences in different error rate.
Table S1 shows the results on the simulated data executed by Euler-SR with k from 19 to 27, and by Euler-mix using Euler-SR as the underlying algorithm. Table S2 shows the results on the simulated data executed by ABySS with k from 19 to 35, and by Euler-mix using ABySS as the underlying algorithm. Table S3 shows the results on the simulated data executed by Edena with k from 19 to 35, and by Euler-mix using Edena as the underlying algorithm.

## Figures and Tables

**Table 1:** Evaluation of assemblies of mouse simulated data with Velvet using different *k*-mer and compare with Euler-mix.

*k*-mer parameter	17	19	21	23	25	27	29	31	33	35	Euler-Mix (19–35)
Precision(overlap)	99.93%	99.98%	99.99%	100.00%	99.99%	99.99%	99.99%	99.99%	99.98%	99.99%	99.99%
Recall(overlap)	9.97%	74.26%	80.27%	79.39%	77.44%	74.22%	69.53%	60.53%	42.08%	7.95%	85.32%
*F*-measure(overlap)	18.14%	85.22%	89.05%	88.51%	87.29%	85.20%	82.02%	75.41%	59.24%	14.73%	92.08%

Precision(consistent)	96.59%	81.29%	93.71%	95.01%	94.50%	93.57%	91.86%	89.02%	85.28%	85.05%	96.31%
Recall(consistent)	8.13%	50.28%	60.73%	60.55%	58.51%	55.28%	50.29%	42.22%	27.58%	4.25%	64.71%
*F*-measure(consistent)	15.00%	62.13%	73.70%	73.96%	72.27%	69.50%	65.00%	57.27%	41.68%	8.09%	77.41%

Number of contigs >= 100 bp	48236	170563	80166	65348	62817	63982	68447	76573	75896	15378	48183
Mean size (bp)	125.28	261.08	582.66	701.67	708.77	663.93	575.11	445.32	306.37	233.43	1001.79
Largest contig	413	3272	11696	27350	45270	81929	81794	27866	14648	8312	81929

N50	121	311	1112	1697	1949	2011	1798	1230	485	250	2783

**Table 2:** Evaluation for assemblies of a real transcriptome dataset of mice with Velvet and compare with Euler-mix.

Assembly pipeline	Number of contigs >= 100bp	Mean size (bp)	Largest contig	N50	# of transcripts detected	Precision (overlap)	Recall (overlap)	F-measure (overlap)	Precision (consistent)	Recall (consistent)	F-measure (consistent)
Velvet (75–21) Velvet (39)	80644	336.32	6668	477	10646	96.44%	39.97%	56.52%	89.82%	22.30%	35.73%
Velvet (33)	106546	191.41	2375	202	2544	97.59%	28.27%	43.85%	86.23%	16.22%	27.31%
