# Supplementary material for: Enhancing De Novo Transcriptome Assembly by Incorporating Multiple Overlap Sizes
Source: ISRN Bioinform. 2012 Apr 23;2012:816402. doi: 10.5402/2012/816402 (PMC4417554; doi:10.5402/2012/816402)
Supplement: Supplementary file 5 [file 816402.f5.pdf]

2 Supplementary Tables S1-S3:

Table S1. Evaluation for assemblies of the mouse simulated data with **EULER-SR** using different *k*-mer and compared with Euler-mix.

k-mer parameter	19	21	23	25	27	Euler-mix (19-27)
Precision (overlap)	99.92%	99.96%	99.97%	99.97%	99.97%	99.96%
Recall (overlap)	68.50%	67.19%	62.83%	62.49%	55.55%	82.20%
F-Measure (overlap)	81.28%	80.37%	77.17%	76.90%	71.42%	90.22%
Precision (consistent)	87.20%	93.33%	94.99%	95.62%	96.06%	90.91%
Recall (consistent)	53.85%	52.20%	48.04%	47.51%	41.53%	63.35%
F-Measure (consistent)	66.58%	66.96%	63.81%	63.48%	57.99%	74.67%
# of contigs >= 100bp	49504	44963	41457	42877	37371	41474
Mean size (bp)	897.56	895.07	877.98	833.26	831.92	1206.58
Largest contig	897.56	80374	81883	81886	81888	81897
N50	2067	2386	2465	2471	2471	2935

Table S2. Evaluation for assemblies of the mouse simulated data with **ABySS** using different *k*-mer and compared with Euler-mix.

k-mer parameter	19	21	23	25	27	29	31	33	35	Euler-mix (19-35)
Precision (overlap)	99.88%	99.97%	100.00%	100.00%	100.00%	100.00%	100.00%	100.00%	100.00%	99.93%
Recall (overlap)	64.50%	75.23%	74.81%	73.79%	71.46%	67.96%	61.99%	50.77%	25.35%	80.28%
F-Measure (overlap)	78.39%	85.85%	85.59%	84.92%	83.36%	80.92%	76.54%	67.34%	40.45%	89.04%
Precision (consistent)	89.35%	96.92%	97.75%	98.06%	98.32%	98.35%	98.34%	97.83%	95.93%	93.89%
Recall (consistent)	48.12%	58.20%	58.35%	57.41%	55.31%	52.19%	47.15%	37.68%	17.47%	61.68%
F-Measure (consistent)	62.55%	72.73%	73.08%	72.42%	70.79%	68.20%	63.74%	54.41%	29.55%	74.45%
# of contigs >= 100bp	63435	33480	32719	33506	32957	33471	35454	39770	33713	28783
Mean size (bp)	609.7	1287.1	1309.34	1254.02	1225.05	1137.78	970.48	695.08	387.77	1638.1
Largest contig	7479	81612	85915	81610	81632	81632	81632	81632	14624	86145
N50	897	2675	2870	2927	2951	2925	2774	2329	1082	3312

Table S3. Evaluation for assemblies of the mouse simulated data with **Edena** using different *k*-mer and compared with Euler-mix-like combined result.

k-mer parameter	19	21	23	25	27	29	31	33	35	Combined result (19-35)
Precision (overlap)	100.00%	100.00%	100.00%	100.00%	100.00%	100.00%	100.00%	100.00%	100.00%	100.00%
Recall (overlap)	76.01%	77.11%	76.54%	74.88%	71.91%	67.02%	58.33%	40.49%	8.02%	81.18%
F-Measure (overlap)	86.37%	87.08%	86.71%	85.64%	83.66%	80.25%	73.68%	57.64%	14.85%	89.61%
Precision (consistent)	86.69%	95.78%	97.17%	97.25%	96.45%	94.48%	91.21%	86.63%	84.98%	98.28%
Recall (consistent)	54.49%	59.48%	59.28%	57.54%	54.44%	49.44%	41.28%	26.74%	4.34%	62.62%
F-Measure (consistent)	66.92%	73.38%	73.64%	72.30%	69.59%	64.91%	56.84%	40.87%	8.25%	76.50%
# of contigs >= 100bp	100177	60541	53532	52720	54858	60379	69107	70239	16538	36094
Mean size (bp)	451.93	738.06	819.47	807.02	739.74	623.25	471.05	316.02	221.63	1267.68
Largest contig	6731	13764	47390	81898	81898	81766	27859	14646	8304	81922
N50	677	1535	1945	2099	2113	1877	1296	512	233	2859
